# Assessment of Black Rot in Oilseed Rape Grown under Climate Change Conditions Using Biochemical Methods and Computer Vision

**DOI:** 10.3390/plants12061322

**Published:** 2023-03-14

**Authors:** Mónica Pineda, Matilde Barón

**Affiliations:** Department of Biochemistry and Molecular and Cell Biology of Plants, Estación Experimental del Zaidín, Consejo Superior de Investigaciones Científicas (CSIC), 18008 Granada, Spain

**Keywords:** artificial intelligence, classifying algorithms, computer vision, deep learning, hyperspectral reflectance, multicolor fluorescence, thermography, *Xanthomonas campestris* pv. *campestris*

## Abstract

Global warming is a challenge for plants and pathogens, involving profound changes in the physiology of both contenders to adapt to the new environmental conditions and to succeed in their interaction. Studies have been conducted on the behavior of oilseed rape plants and two races (1 and 4) of the bacterium *Xanthomonas campestris* pv. *campestris* (Xcc) and their interaction to anticipate our response in the possible future climate. Symptoms caused by both races of Xcc were very similar to each other under any climatic condition assayed, although the bacterial count from infected leaves differed for each race. Climate change caused an earlier onset of Xcc symptoms by at least 3 days, linked to oxidative stress and a change in pigment composition. Xcc infection aggravated the leaf senescence already induced by climate change. To identify Xcc-infected plants early under any climatic condition, four classifying algorithms were trained with parameters obtained from the images of green fluorescence, two vegetation indices and thermography recorded on Xcc-symptomless leaves. Classification accuracies were above 0.85 out of 1.0 in all cases, with k-nearest neighbor analysis and support vector machines performing best under the tested climatic conditions.

## 1. Introduction

Climate change is one of the most pressing problems facing humanity. Particularly, the agriculture industry is an alarming sector on a global scale, as irreversible weather fluctuations threaten food quality, production, and supply [1]. As a consequence of this direct negative impact on the main source of human food, governments and supra-state institutions have organized themselves to analyze the consequences of climate change and, ultimately, to try to curb it. The United Nations established the Intergovernmental Panel on Climate Change (IPCC) to evaluate potential scenarios of greenhouse gas and air pollutant emissions and their future concentrations in the atmosphere, publishing Assessment Reports (AR) containing these projections. Based on IPCC estimates, future climate conditions have been anticipated according to representative concentration pathways (RCPs) [2]. The main objective of the Paris Agreement [3], promoted by the United Nations and recently ratified by the Sharm el-Sheikh Climate Change Conference (Egypt, 2022), is to improve the global response to the urgent threat of climate change and to join efforts to limit the temperature increase to 1.5 °C by 2080–2100. However, according to AR5, this stringent mitigation scenario is unlikely to occur, and the most likely projection is an intermediate scenario or RCP 4.5 in which temperature would increase by 3 °C. An extreme scenario of climate change or RCP 8.5 would represent the effects of no further restriction of greenhouse gas emissions and would result in a 6 °C increase in global temperature [2].

Climate change causes serious physiological disturbances in plants by impairing their growth, disrupting photosynthesis, and reducing their ability to respond to stress [4]. However, another problem associated with climate change is that its effects will not be limited to direct damage to crops, but pathogens and pests (both endemic and newly emerging) may be exacerbated by global warming [5]. Future climate will affect, among other factors, the physiology of pathogens and pests and their geographical distribution [6,7], thus acting as evolutionary forces that could alter their degree of virulence [8]. Therefore, scientific innovation is very necessary for the early detection of diseased plants to minimize the risk of disease spread and to avoid the introduction of new ones under current climate conditions (CCC), but without losing sight of the challenges associated with climate change [9,10]. In this regard, the development of new technologies, such as high-performance sensors for pathogen detection together with artificial intelligence (AI) tools, have enabled disease surveillance, localizing the foci of infection and thus promoting precision agriculture [11].

In the last years, non-invasive optical sensors have been increasingly implemented to monitor crop fields at proximal and remote sensing scales [12,13]. Thermal imaging provides information on leaf temperature (T), which is inversely related to leaf transpiration, and thus it has been used with great success to assess the crops′ water status [14]. Multicolor fluorescence imaging (MCFI) records blue (F440) and green (F520) fluorescence emitted by phenolic compounds related to plant defense and covalently bound to cell walls [15]. Measurements of reflected light at different wavelengths allow the calculation of vegetation indices (VIs) widely used to evaluate different vegetation traits such as development, productivity, pigment composition, and fitness [16]. Nevertheless, imaging techniques also have the disadvantage of properly handling the vast and complex amount of information that could be obtained from images. Deep learning, a subset of AI, has shown great potential to help the agricultural industry due to the powerful ability of its algorithms to learn features from massive datasets and make predictions about new samples [17], thus assisting in the monitoring and decision-making processes of crop management [18]. There is a wide variety of algorithms for the detection of stressed plants. Among the most broadly used ones stand out artificial neural networks (ANN), support vector machines (SVM), binary logistic regression analysis (LRA), and k-nearest neighbors analysis (kNN). ANNs are networks based on the human nervous system, thus composed of several interconnected nodes (or neurons) organized in layers that transmit information to each other, learning from both input and output data. ANNs have been frequently applied in the detection of stressed plants, as they obtain very reliable results [19,20]. SVM represents samples as points in a high-dimensional feature space defined by support vectors, and new data are predicted to belong to a category based on the side of the hyperplanes on which they fall. The more advanced machine learning technique of SVMs uses a larger number of hidden units than ANNs and thus performs better in formulating the learning problem [19,21]. LRA is particularly interesting for the life sciences, as it allows estimating the probability of a dichotomous outcome (“healthy” or “infected”) as a function of the values of one or more independent variables [22]. Finally, kNN assigns relative weights to the contributions of the sample neighbors as a function of distances to determine to which category a new sample is most likely to belong [23]. Computer vision and deep learning algorithms have been widely applied in precision agriculture in recent years ([24,25] and references therein). ANNs have been used to detect oilseed rape plants infected by *Sclerotinia sclerotiorum* [26] or zucchini plants infected by *Dickeya dadantii* [27]; SVMs were useful to detect Huanglongbing disease on citrus leaves [28] or zucchini plants infected with *Podosphaera xanthii* [29]; LRA was the best performing algorithm for identifying avocado trees infected by *Rosellinia necatrix* [30]; whereas kNN was successfully used to identify tomato leaves diseased by gray mold [31], to cite just a few of the many examples.

Oilseed rape (*Brassica napus* L.) is the main oilseed crop in Europe. This crop is not only a source of oil for human consumption but also provides feed for livestock and can even be used as biodiesel, among other uses [32,33]. With 5.17 million hectares of oilseed rape cultivated in the European Union (EU) in 2020 and being a crop in expansion in some countries such as Spain (according to data from DG AGRI EU for the Spanish Ministry of Agriculture, Fishing and Food; www.mapa.gob.es, accessed on 21 December 2022), it is not surprising that the response of this crop-to-climate change has been studied on several occasions. Thus, abiotic stress triggered by future weather conditions has been demonstrated to damage photosynthetic structures [34,35,36], thus reducing crop growth and production, as well as yield quality, mainly oil properties [37,38,39]. In addition, oilseed rape is a plant susceptible to black rot of crucifers, an infection caused by the pathogenic bacterium *Xanthomonas campestris* pv. *campestris* (Xcc), which is responsible for severe economic losses in crops worldwide [40,41]. Infected seeds are the main source of Xcc infection, although this bacterium can also live epi- and endophytically in volunteer plants, crop debris, and even soils. Xcc penetrates the plant cell wall through wounds and natural openings such as stomata, hydatodes, and lenticels, colonizing the mesophyll [42,43,44]. The infection becomes systemic when Xcc reaches the xylem, plugging it and preventing water and nutrients from reaching it. This results in the appearance of characteristic V-shaped patterns in leaf margins, initially chlorotic and eventually necrotic, as the infection progresses from biotrophic to necrotrophic stages. Eventually, plants die [45,46,47]. In addition, it is thought that Xcc will become an increasingly important disease due to climate change in the more northern latitudes of vegetable production, including the warmer regions of Europe [42]. Moreover, the global change would accelerate the Xcc infection progress, at least in broccoli plants [48]; however, more research is needed, as little is known about the alterations that will occur at the physiological level of Xcc-infected oilseed rape plants growing under global warming. It would also be advisable to be prepared in terms of early detection of Xcc-infected oilseed rape plants under any climatic condition in order to anticipate our response now and in the near future adequately.

Thus, the main objectives of this work were twofold. The first one is related to the effects that two possible climate change scenarios for 2100 would produce on the health status of Xcc-infected oilseed rape plants. For such a purpose, oilseed rape plants were cultivated under CCC, RCP 4.5, and RCP 8.5 and infected with races 1 and 4 of Xcc. Then, visual symptoms of Xcc-infected plants, plant oxidative stress, and plant pigment composition was analyzed by colorimetric biochemical methods. The effects of climate change on Xcc growth were also tested by analyzing bacterial growth kinetics both in vivo and in vitro. Evidence was found that climate change harms both plants and bacteria, but Xcc benefits from the interaction, and its infection accelerates senescence already induced by climatic stress. The second objective was to develop a method for early detection of Xcc-infected oilseed rape plants based on computer vision and deep learning algorithms that were effective regardless of weather conditions. For this purpose, images of F520, T, and two VIs from asymptomatic Xcc-infected and mock-control leaves were recorded. Inoculated areas were then selected, and pixel value distributions were obtained for each image, as well as the parameters defining those histograms. Parameters significantly different between mock-control and Xcc-infected leaves were selected to train four classifiers (ANN, SVM, LRA, and kNN) at each climatic condition tested. The obtained results indicate that computer vision and AI classification algorithms have the ability to detect Xcc infections in oilseed rape plants at an early stage. Additionally, the significance of taking into account the impact of environmental conditions on the performance of the classification of these methods is emphasized.

## 2. Results

### 2.1. Development of Symptoms Induced by the Biological and Climatic Treatments Tested

**Figure 1:**
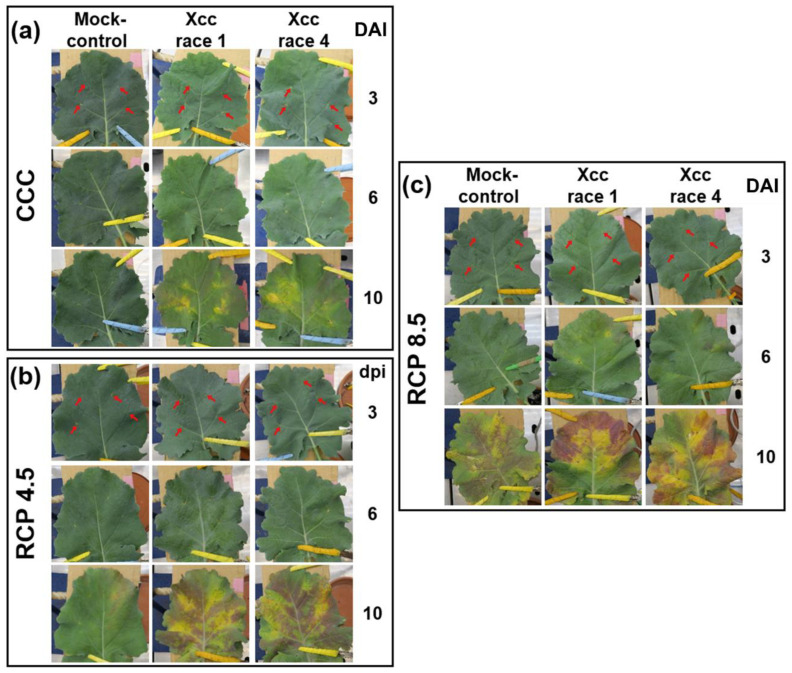
Symptoms developed by the oilseed rape leaves upon the three biological treatments (mock-control, *Xanthomonas campestris* pv. *campestris* (Xcc) race 1-, and Xcc race 4-infected leaves) when cultivated under the three climatic treatments assessed in this work: (**a**) current climatic conditions (CCC); (**b**,**c**) representative concentration pathways (RCP) 4.5, and 8.5, respectively. Red arrows indicate the points where the bacteria were inoculated at doses of 10^8^ cfu·mL^−1^ on the fourth leaf of oilseed rape plants. Leaves are shown at 3, 6, and 10 days after inoculation (DAI).

The fourth leaf of oilseed rape plants grown under each climatic treatment was inoculated (Xcc race 1 or 4, or mock-inoculated) by clipping in four secondary veins, and symptoms were followed up to 10 days after inoculation (DAI) (Figure 1). At CCC, mock-control leaves did not undergo any symptoms except for the dryness of the inoculated tissues, as did leaves inoculated with Xcc from 3 DAI. However, leaves of plants inoculated with both Xcc races developed chlorosis of the tissue surrounding the inoculation point on successive days, being clearly pronounced at 10 DAI (Figure 1a).

Climate change triggered abiotic stress to mock-control leaves that was more severe when the climatic treatment imposed was more intense. Whole leaves showed very slight chlorosis at 6 DAI that progressed thereafter. At 10 DAI, mock controls also showed reddish-purple pigments. In the case of Xcc-infected leaves, biotic stress introduced additional damage to that caused by abiotic stress. Mild (RCP 4.5; Figure 1b) or evident (RCP 8.5; Figure 1c) chlorosis surrounded the inoculation points as early as 6 DAI. In those plants growing at RCP 8.5, it was also possible to find reddish spots near inoculation sites at this time point. Chlorosis evolved with a characteristic V-shape, which eventually led to necrosis in those plants growing at RCP 8.5. The gaps between the chlorotic zones appeared deep reddish-purple.

It is worth mentioning that the symptoms caused by both races of Xcc on the leaves of oilseed rape plants were indistinguishable, to the naked eye, in any of the climatic treatments tested.

### 2.2. Growth of Races 1 and 4 of Xanthomonas campestris pv. campestris on Oilseed Rape Leaves from Plants Cultivated under Each Climatic Treatment

**Figure 2:**
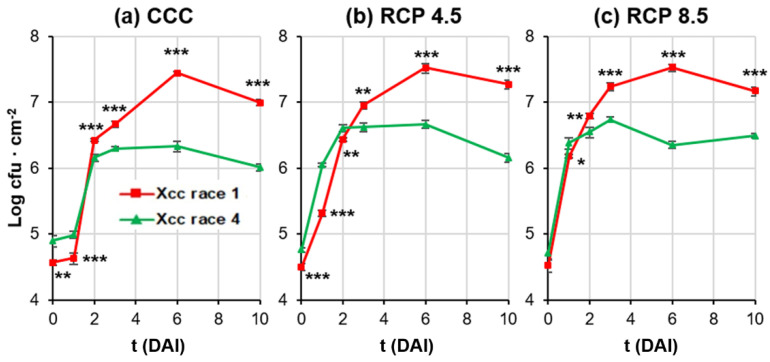
Growth kinetics of Xcc in the circular area surrounding the inoculation point of oilseed rape leaves, measured as colony forming units per square centimeter (cfu · cm^−2^) from two hours after inoculation until 10 days after inoculation (DAI). Bacterial density found on leaves of plants grown under CCC (**a**), RCP 4.5 (**b**), and RCP 8.5 (**c**). Data represent means ± standard error of six leaf disks. Significant differences between biological treatments are indicated by asterisks at *p* < 0.05 (*), *p* < 0.01 (**), and *p* < 0.001 (***).

Xcc growth was followed in leaf zones concentric to the inoculation point from two hours after infection until 10 DAI (Figure 2). Growth during the log phase was faster for race 4 than for race 1 (excepting for RCP 8.5), reaching the plateau at 2 DAI, whereas race 1 rose the stationary phase later, at 3 (RCP 8.5) or 6 DAI (CCC and RCP 4.5). On the other hand, the longer log phase of race 1 caused this race to reach the highest concentration at 10 DAI, registering 10 times more colony forming units per square centimeter (cfu · cm^−2^) than race 4 in each climatic treatment tested. RCP 8.5 treatment accelerated the growth of both races of Xcc, as demonstrated by the higher slope in the log phase; in addition, a higher number of cfu · cm^−2^ was recovered from Xcc race 4 at the stationary state.

Bacterial growth was also tested in vitro for two days. Growth conditions reproduced those used for each climatic treatment at which plants were grown (Figure S1). Compared to CCC, RCP 4.5 induced a shorter log phase for both bacteria, as well as a faster growth of race 4 with respect to race 1; however, the number of cfu per milliliter recovered during the plateau phase was very similar for both races and climatic treatment. On the other hand, no bacteria could be recovered when cultures were growing under RCP 8.5 conditions.

### 2.3. Measurements of Oxidative Stress and Pigment Content in Leaves of Oilseed Rape Plants Grown under the Biological and Climatic Treatments Assayed

**Figure 3:**
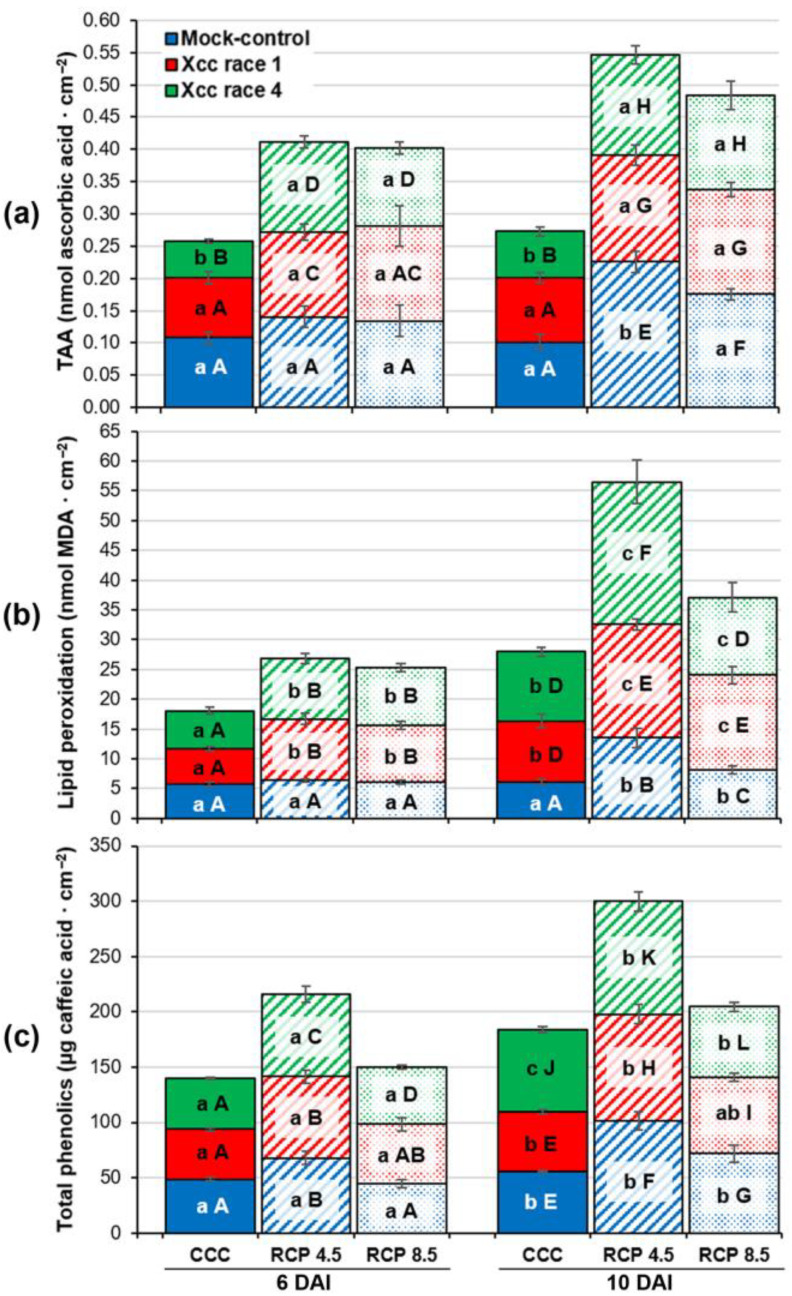
Determination of: (**a**) total antioxidant activity (TAA), (**b**) lipid peroxidation, and (**c**) total phenolic content in the fourth leaf of oilseed rape plants at 6 and 10 days after inoculation (DAI). Graphs show means ± standard error of six leaf disks sampled from six plants. Blue, red, and green colors represent each biological treatment (mock-controls; Xcc race 1-, and Xcc race 4-infected leaves, respectively), whereas solid-colored, striped, and dotted bars show the results obtained for each climatic treatment (CCC, RCP 4.5, and RCP 8.5, respectively). For each climatic treatment, different lowercase letters indicate significance between biological treatments (*p* < 0.05). In contrast, for each biological treatment, different uppercase letters show significance between climatic treatments (*p* < 0.05). Consequently, lowercase letters should be interpreted vertically, while the interpretation of uppercase letters should be made horizontally.

To assess the oxidative stress that biological and climatic treatments caused to oilseed rape leaves, total antioxidant activity (TAA), lipid peroxidation, and total soluble phenolic content were quantified (Figure 3). TAA measures the ability of antioxidant substances in leaves to counteract free reactive oxygen species (ROS); lipid peroxidation is a marker of oxidative stress; and several phenolic compounds are known to have antioxidant activity.

Climate change (both RCP 4.5 and 8.5) imposed abiotic stress to mock-control oilseed rape leaves that led to higher TAA at 10 DAI relative to CCC, as well as higher total phenolic content at 6 DAI (RCP 4.5) or/and 10 DAI (RCP 4.5 and RCP 8.5). In spite of this increase in antioxidant substances, both climate change treatments induced lipid peroxidation at 10 DAI.

Regarding biological treatments at CCC, race 4 of Xcc caused a decrease in the TAA of infected leaves at 6 and 10 DAI, whereas race 1-infected leaves did not show significant changes respecting the mock-controls. The consequence of this reduction in TAA was an increase in lipid peroxidation of infected leaves relative to mock controls at 10 DAI, which was also registered in leaves infected with Xcc race 1. Total soluble phenolics were also increased in leaves infected by Xcc race 4 at 10 DAI but not in those infected with race 1.

Xcc infection added a biotic component to the abiotic stress already caused by climate change. At RCP 4.5, Xcc-infected plants showed lower TAA than the controls at 10 DAI and increases in lipid peroxidation from 6 DAI. However, no significant differences in soluble phenolic content could be found between mock-controls and Xcc-infected leaves at any DAI assayed. On the other hand, the three biological treatments showed similar TAA and soluble phenolic content at RCP 8.5, whereas lipid peroxidation of Xcc-infected plants took place as early as 6 DAI.

In general terms, alterations caused by races 1 and 4 of Xcc on TAA, lipid peroxidation, and total soluble phenolic content of oilseed rape leaves were very similar. In fact, no significant differences could be found between leaves inoculated with race 1 and those infected with race 4 at RCP 4.5 and RCP 8.5. Contrarily, significant differences were registered between leaves infected with both races of Xcc in TAA (from 6 DAI) and soluble phenolic content (at 10 DAI) but not in lipid peroxidation at CCC.

**Figure 4:**
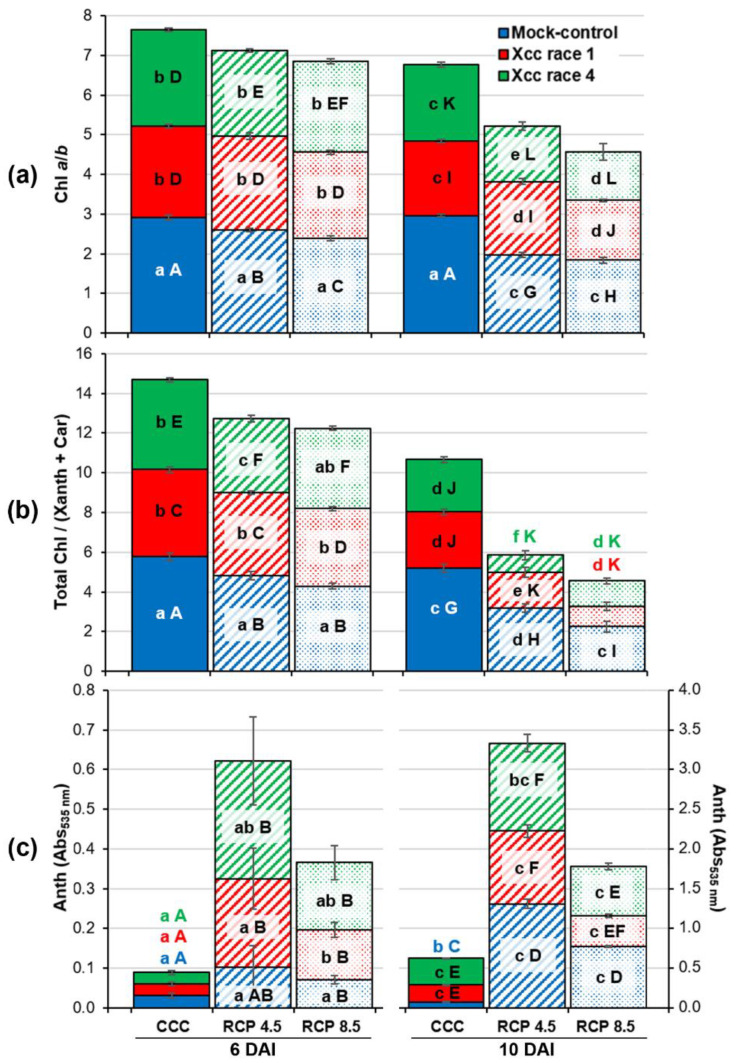
Measurements of: (**a**) the ratio of chlorophyll *a* over chlorophyll *b* (Chl *a*/*b*), (**b**) the ratio of total chlorophyll content over the xanthophylls and carotenoids content (Total Chl/(Xanth + Car)), and (**c**) anthocyanins content (Anth) in the fourth leaf of oilseed rape plants at 6 and 10 days after inoculation (DAI). Graphs show means ± standard error of six leaf disks sampled from six plants. Bars features (colors, patterns) and well as lower and uppercase letters should be interpreted as in Figure 3. Please, note that the scale of the graph shown in (**c**) is different for 6 than for 10 DAI.

Pigment composition was also analyzed based on the color changes observed in oilseed rape leaves. The progression of chlorosis and the appearance of red-purple pigments can be quantified by measuring the Chl *a*/*b* (chlorophyll *a*/*b*) ratio, the total Chl amount over the sum of Xanth (xanthophylls) and Car (carotenoids), and the content of Anth (anthocyanins; Figure 4).

As a consequence of climate change, mock-control leaves showed decreases in the Chl *a*/*b* and total Chl/(Xanth + Car) ratios respecting the levels registered for CCC from 6 DAI, which were more pronounced for plants growing at RCP 8.5. On the other hand, Anth increased considerably at 10 DAI as a consequence of climate change.

With respect to biological treatments, Xcc infection reduced the Chl *a*/*b* and total Chl/(Xanth + Car) ratios from 6 DAI at each climatic treatment assayed. Races 1 and 4 induced similar decreases with respect to the mock controls, except for those leaves of plants growing at RCP 4.5 at 10 DAI, where race 4 induced a greater decline than race 1. At CCC, but not in RCP 4.5 and RCP 8.5, Anth levels increased as a consequence of the infection with both races of Xcc at 10 DAI.

### 2.4. Computer Vision Coupled to Classifying Algorithms to Identify Healthy and Xanthomonas campestris pv. campestris-Infected Leaves

To identify Xcc-infected plants at an early stage by deep learning algorithms, the fourth leaf of oilseed rape plants were imaged by MCFI, thermography, and hyperspectral reflectance cameras before the Xcc symptoms appearance: 6 DAI for CCC and 3 DAI for RCP 4.5 and RCP 8.5. To feed classifying algorithms, it is crucial to obtain parameters from images that unambiguously define categories to be classified. Thus, the first task was to identify those parameters that would best show the differences between mock-control leaves and those infected with both races of Xcc. The workflow will be described using F520 images as an example, but it was extensible to the rest of the images taken.

**Figure 5:**
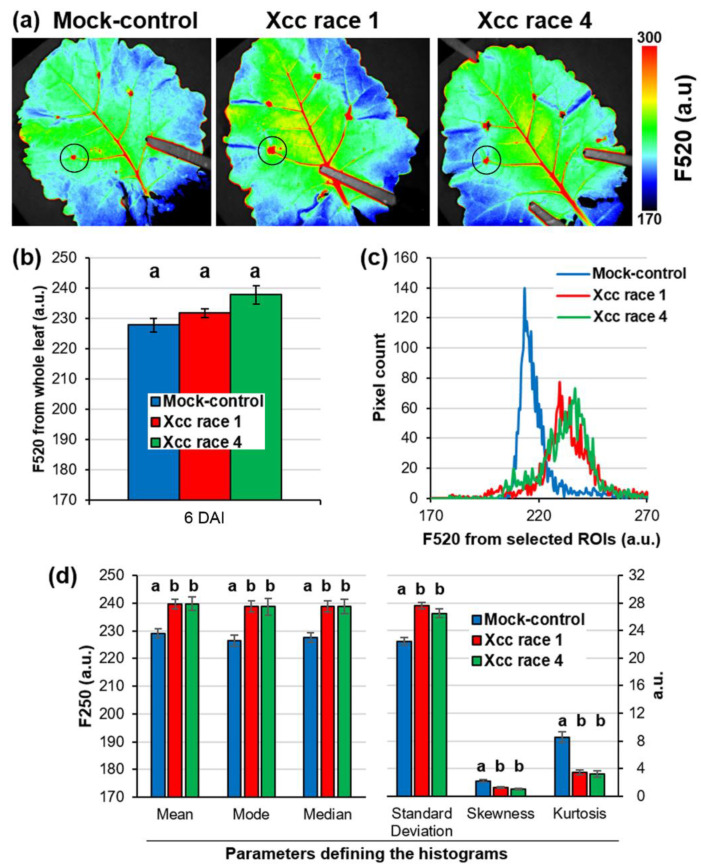
(**a**) Images of green fluorescence (F520) emitted by representative mock-control, Xcc race 1-, and race 4-infected oilseed rape leaves of plants grown under CCC at 6 days after inoculation (DAI). Black circles indicate selected regions of interest (ROIs) concentric to inoculation points. (**b**) F520 emission averaged for the whole leaf. Graphs show the mean ± standard error of 11 leaves per biological treatment. (**c**) Histograms showing the distribution of F520 pixel values of the ROIs indicated in (**a**). (**d**) Parameters defining the histograms. Graphs display the mean ± standard error of 44 ROIs per biological treatment. Lowercase letters show significant differences at *p* < 0.05.

To simplify the work, whole leaf images were used at first, as they were easy to isolate from the image background. Figure 5a shows the F520 emission of leaves of plants grown at CCC at 6 DAI. Main veins, as well as inoculation points, appeared red, indicating higher F520 emission. However, this increased F520 level was restricted to areas affected by the mechanical damage caused by the tweezers in the case of mock-control leaves, whereas the area affected by Xcc infection was wider. Moreover, some yellow pixels appeared outside the main veins on Xcc-infected leaves, while they were not visible on mock-control leaves. Despite this, when averaging the F520 registered across the whole leaves, no significant differences were found between mock-control and Xcc-infected leaves (Figure 5b). The offset between high and low pixel values of a parameter when averaging whole leaf measurements that resulted in a loss of spatial information not only occurred with F520 but was common to most of the parameters recorded by sensors.

Therefore, two decisions were made to work with images. The first was to use regions of interest (ROIs) concentric to the inoculation point instead of entire leaves, as these were the areas where the greatest changes between biological treatments were concentrated. In addition, by having four inoculation points per leaf, the sample size was multiplied by four. The second decision was to work with histograms displaying the distributions of pixel values instead of simple averages to avoid loss of spatial information. Thus, Figure 5c displays the histograms of the ROIs drawn in Figure 5a. The pixel value distributions of the F520 corresponding to ROIs of Xcc-infected leaves were very different in width and height to the ROIs of the control leaf, as well as being shifted to the right. Those differences can be quantified using parameters describing histograms: maximum, minimum, extent = maximum − minimum, mean, mode, median, standard deviation, skewness, and kurtosis. Several of these parameters defining histograms were significantly different (*p* < 0.05) between mock-control and Xcc-infected leaves but not between leaves of plants infected with races 1 and 4 (Figure 5d). This was true not only for F520 but also for many other parameters registered by imaging techniques. Therefore, it seemed reasonable to work with only two categories of leaves when classifying them: “healthy” (or mock-controls) and “Xcc-infected”.

Thus, for each climatic condition and before the onset of the symptoms, histograms from ROIs of F520, T, water balance index (WBI), and disease broccoli index number 3 (DBI_3_) images were obtained, as these images showed a spatial pattern characteristic for Xcc infection. The parameters defining the histograms were then calculated and compared between the three biological treatments for each climatic condition using a two-tailed Student *t*-test. Only those parameters that showed significant differences between healthy and Xcc-infected leaves at *p* < 0.05 at each climatic treatment were incorporated into a database to be implemented in classifying algorithms (Table 1).

**Figure 6:**
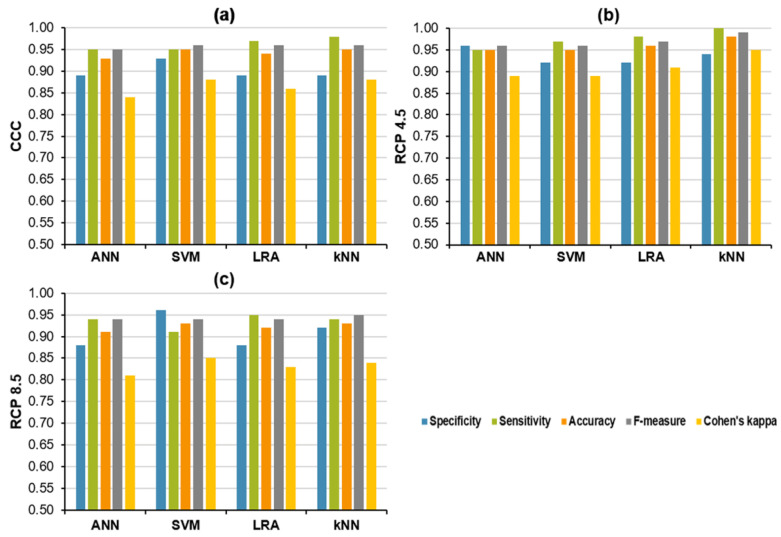
Results of the cross-validation of the four algorithms used to classify healthy and Xcc-infected oilseed rape leaves for each climatic treatment: (**a**) CCC, (**b**) RCP 4.5, and (**c**) RCP 8.5. ANN: multilayer perceptron based-artificial neural networks; SVM: support vector machines; LRA: logistic regression analysis; kNN: k-nearest neighbors analysis.

The classification performances of four algorithms were tested in each climatic treatment: ANN, SVM, LRA, and kNN. All the fitted models successfully classified healthy and Xcc-infected leaves in any climatic treatment. The ability to correctly identify healthy leaves (specificity) and Xcc-infected leaves (sensitivity), as well as the capacity to make right guesses (overall accuracy), was always higher than 0.85 out of 1 in all cases, i.e., for the four models and the three climatic treatments (Figure 6).

For a better evaluation of the fitted models, other statistics have been computed. F-measure is a way of combining precision and sensitivity into a single harmonic calculation that captures the properties of both to return a more general measure of model quality. On the other hand, Cohen’s kappa indicates the proportion of correct classifications that are not due to chance. F-measure and Cohen’s kappa can take values between 0–1, and the performance of classifications of the fitted models will be better the closer these statistics are to 1. Following these criteria, SVM and kNN were the algorithms with the best classification performance at CCC (Figure 6a), obtaining the highest scores in the five statistics evaluated, whereas kNN would be the most successful algorithm at RCP 4.5 (Figure 6b). Concerning RCP 8.5, the classification performance of the four algorithms worsened, and the chance hit capacity increased, as indicated by the lower Cohen’s kappa values. SVM and kNN rendered a similar classification performance that outperformed the other models at RCP 8.5 (Figure 6c).

## 3. Discussion

Climate change could have a significant impact on plant diseases depending on whether environmental conditions are conducive to diseases [49]. It is known that oilseed rape plants grown under climate change conditions underwent premature leaf senescence that is directly proportional to the severity of the climatic treatment imposed [34]. Thus, this premature senescence experienced by plants growing under RCP 4.5 and especially RCP 8.5 climatic treatments may have made them more vulnerable to Xcc infection, as evidenced by earlier onset and worsening of symptoms observed in these plants compared to those grown under CCC. Higher disease severity associated with increased ambient temperatures was also found in other plants [50,51,52]. In turn, increased CO_2_ levels have different impacts on pathogen severity depending on the host–pathogen system analyzed: it can lower the pathogen incidence [53], or contrarily, it can raise the pathogen aggressiveness [54,55,56]. However, the combination of elevated temperature and CO_2_, which is expected to occur due to climate change, increases the severity of numerous plant diseases, as reviewed by [57].

To understand the mechanisms underlying Xcc infection in oilseed rape plant metabolism and how they may be influenced by climate change, experiments to measure oxidative stress and pigment composition were conducted. The imbalance between the production of ROS and antioxidant capacity causes oxidative stress leading to damage to cellular components, including thylakoid membranes, and thus modifies the pigment composition of chloroplasts [58]. Oxidative stress was evaluated in terms of TAA, lipid peroxidation, and total phenolic content. TAA measures the capacity of antioxidant substances present in leaf samples to scavenge free ROS, whereas lipid peroxidation is a marker of oxidative stress [34]. Xcc infection, especially that produced by race 4, reduced the TAA and increased lipid peroxidation in the leaves of plants grown under CCC. This is in agreement with other studies that also measured decreases in TAA [59,60], increases in lipid peroxidation [61,62,63], or both [64] in plants upon pathogen infections. Regarding climatic treatments, mock-control plants grown under RCP 4.5 and RCP 8.5 displayed an increase in TAA at 10 DAI that, in turn, could not prevent plants from suffering damages in membranes, as measured by the increment in lipid peroxidation with respect to plants grown under CCC at this time point. Lipid peroxidation is thought to be responsible for the premature leaf senescence observed in plants grown under climate change conditions [34]. Thus, Xcc enhanced and accelerated the oxidative stress already triggered by climate change, as lipid peroxidation could be detected in infected plants as early as 6 DAI, reinforcing the hypothesis that climate change increases the oilseed rape plants’ susceptibility to Xcc. On the other hand, phenolic compounds synthesized by secondary plant metabolism are involved in the defense of plants against stress due to their antioxidant properties, among other functions [65,66,67]. Climatic treatments RCP 4.5 and RCP 8.5 induced slight increments in soluble phenolic content of mock controls, probably to try to counteract the negative effects of oxidative stress [34]. However, there was no correlation between the severity of Xcc infection and the measured soluble phenolic content in any of the climatic treatments tested. This seems to be the general trend in the brassica’s response to Xcc [68]. Indeed, it has been suggested that secondary metabolism would play a less prominent role in defense against Xcc in cabbage plants (another brassica) than primary metabolism [69].

Chlorosis is a marker of leaf senescence, a process associated with leaf aging but which can also be induced by environmental stress [70,71], such as elevated temperatures and high atmospheric CO_2_ levels [72,73,74]. Moreover, the composition and content of leaf pigments change in plants infected with pathogens that induce chlorotic and necrotic symptoms [75], probably due to lipid peroxidation-induced breakdown of thylakoid membranes [76]. In healthy leaves, the Chl *a*/*b* ratio is fine-tuned to ensure the correct development of photosynthesis; however, Chl *b* is less sensitive to oxidative stress than Chl *a*. Thus, it is frequent to find decreases in the Chl *a*/*b* ratio in stressed plants [77]. Declines in the total Chl/(Xanth + Car) ratio are also common in chlorotic plants, as Xanth and Car catabolism occurs to a lesser extent than Chl degradation [78,79,80]. Mock-control oilseed rape plants grown under climate change treatments registered decreases in both ratios, with respect to those grown under CCC that was proportional to the severity of the imposed climate change projection. Therefore, climate change accelerates the appearance of chlorosis (and thus, the senescence process) in leaves of oilseed rape plants as a consequence of membrane damage induced by lipid peroxidation [34]. On the other hand, Xcc infection also unbalanced the pigment composition of infected plants, as indicated by lower measured values of Chl *a*/*b* and total Chl/(Xanth + Car) ratios, as in the case of rice plants infected with *Xanthomonas oryzae* pv. *oryzae* [81] and tomato leaves infected with *X. campestris* pv. *vesicatoria* [82]. The cumulative effects of climate change and bacterial infection caused oilseed rape plants growing under RCP 8.5 to be most affected by oxidative stress. In contrast, the effects of climatic and biological treatments were not cumulative in the case of Anth. These red-purple pigments are involved in abiotic stress tolerance and resistance to herbivores and pathogens, helping plants to scavenge free ROS, among other functions [83,84]. Anth is also synthesized during leaf senescence when Chl degradation makes them more noticeable [85]. Thus, mock-control oilseed rape plants produced more Anth when grown under RCP 4.5 and RCP 8.5 with respect to those cultivated under CCC, probably as a part of a strategy to avoid lipid peroxidation [34,86]. On the other hand, Xcc infection produced an increment in Anth content when plants were grown under CCC, as in the case of apple trees infected with cedar-apple rust [87]. However, the combined effect of climate change and bacterial infection did not enhance the accumulation of Anth in infected leaves. The only notable feature was the different visual pattern of Anth accumulation between mock-controls and Xcc-infected leaves. The mock controls did not show a specific pattern of Anth distribution, accumulating throughout the whole leaf. Instead, leaves of Xcc-infected plants accumulated Anth mainly in the areas surrounding the V-shaped lesions, probably in an attempt to alleviate the symptoms caused by the spreading bacteria. The movement of the *Brassica yellows virus* also caused the accumulation of Anth during its spreading through tomato plants [88].

Interestingly, visual symptoms caused by biological treatments in oilseed rape plants did not differ from one Xcc race to another under any climatic condition, unlike broccoli [48] or Arabidopsis [89] plants, where it was possible to see differences in symptoms caused by races 1 and 4. This could compromise the ability of the classifying algorithms to distinguish plants infected with both races, as will be discussed later. Furthermore, the lack of significant differences between the two races of Xcc in most of the physiological parameters measured (regardless of climatic treatment assayed) seems to be in line with the absence of differences in symptoms caused by both races. This was true even though the number of bacteria recovered from live tissues was clearly different for both races. From the growth graphs it could be deduced that a minimum concentration above 10^6^ cfu · cm^−2^ is sufficient to produce symptoms and that, thereafter, the number of cfus is not proportional to the degree of symptomatology. As this critical concentration is reached earlier under climate change conditions, especially under RCP 8.5 treatment, the onset of symptoms is consequently brought forward. Moreover, physiological alterations induced by climate change could make plants more vulnerable to Xcc, as discussed above. Additionally, the bacterium could also be favored by higher temperatures and increased levels of atmospheric CO_2_ [51]. To test this hypothesis, races 1 and 4 of Xcc were grown in vitro under the three climatic treatments. Both races grow similarly in vitro, except under RCP 8.5, where no live cells could be recovered. Thus, the plant seems to make a difference for Xcc growth, and the epiphytic subsistence of this bacterium could be threatened under RCP 8.5 growth conditions, which could instead favor the crops.

Overall, these findings highlight the complex interplay between the bacterial pathogen, the host plant, and the environment in determining the outcome of an infection. However, it can be concluded that climate change may exacerbate the negative effects of Xcc infection on oilseed rape plants. This information could be useful in the development of strategies to mitigate the negative impacts of pathogen infection on crops and to adapt to the challenges of climate change in agriculture.

The second objective of this work was to develop an effective method for the detection of Xcc-infected oilseed rape plants based on computer vision and deep learning algorithms in each climatic condition. This method has been used with great success in the last few years due to the reduction in the cost of image sensors and the development of deep-learning classification methods [90,91,92]. In this work, thermal, MCFI, and hyperspectral reflectance measurements of mock-control and Xcc-infected oilseed rape leaves were taken using imaging sensors. To ensure the identification of Xcc-infected plants at an early stage, measurements were performed just before the symptoms appeared in each climatic condition (6 DAI for CCC and 3 DAI for RCP 4.5 and RCP 8.5). Histograms from areas surrounding the inoculated points were used to avoid the offset between high and low pixel values when averaging whole leaf measurements. Histograms showing the frequency of pixel values for a parameter of interest are convenient tools to find differences between treatments [93], and the parameters defining those histograms have been previously shown to be useful to feed algorithms able to detect bacterial and fungal infections on cucurbits [29]. Thus, histograms of ROIs were extracted from F520, T, WBI, and DBI_3_ images. Not surprisingly, images of these parameters revealed a characteristic spatial pattern for Xcc-infected leaves that images of leaves from mock-control plants did not show. F520 comes from defensive phenolic compounds covalently bound to cell walls [15], and plants may be accumulating them in an attempt to contain the bacterial spreading. Xcc is known to plug the xylem vessels [94], and parameters such as T and WBI are sensitive to changes in leaf water status [14,95]. Moreover, DBI_3_ (a VI that was specifically designed to detect Xcc infection on broccoli plants [48]) is related to lipid peroxidation [34]. Relevant parameters describing histograms were incorporated into three databases, one for each climatic treatment. Data were organized into two categories: “healthy” and “Xcc-infected”, as it was not possible to find differences between leaves of plants infected with both races of Xcc. This is consistent with the lack of differences in most of the measured physiological parameters, as well as visual symptoms, between Xcc race 1 and race 4 treatments, as discussed above. Distinguishing between plants infected by one race or another is irrelevant in practice since treatments against Xcc do not depend on the infecting race.

The classification performance of all tested algorithms was very successful in every climatic treatment, demonstrating the effectiveness of the chosen method. For plants grown under CCC, the classification performance of ANN, SVM, LRA, and kNN at CCC were similar in terms of the five indicators evaluated. However, SVM and kNN performed best since they showed the higher F-measure and Cohen’s kappa values. Nevertheless, kNN displayed slightly higher sensitivity than SVM and, therefore, low false negative rates, which can be considered more convenient when detecting diseased plants [30,96]. These results were comparable to that recently reported by Zhang et al. [97], who found that convolutional neural networks were able to detect peach leaf disease produced by *X. campestris* with 100% accuracy, and to those informed by Shahoveisi et al. [26], who reported accuracies above 89% when applying ANNs to the detection of tomato or oilseed rape plants infected by *Sclerotinia sclerotiorum*. On the other hand, the model described here considerably outperformed the maximum mean average precision (56.9%) obtained when different classifying algorithms were applied to detect aphid colonies in *Brassica napus* crops [98].

According to previous results reported for broccoli plants growing under climate change conditions and infected by races 1 and 4 of Xcc [48], the performance of the classifying models was affected differentially by growing conditions. Whereas the classification capability of the algorithms may increase slightly for oilseed rape plants grown at RCP 4.5, the predictive ability would decrease if the worst predictions of climate change (RCP 8.5) were to come true, as lower Cohen′s kappa values showed. However, the decrease in the predictive capability of the models developed for oilseed rape was not as marked as that recorded for the broccoli models. Thus, the approach employed in this work (use of ROIs and histograms, classification into two categories: “healthy” and “Xcc infected”), although more laborious, seems to be more efficient compared to that used for broccoli (parameters averaged for whole leaves, classification into three categories: “healthy”, “Xcc race 1-infected“ and “Xcc race 4-infected”). As in the case of plants grown under CCC, kNN would be the model of choice, followed by SVM, in the projected future climate conditions.

In conclusion, climate change has a significant impact on Xcc infection in oilseed rape plants, as premature senescence caused by elevated temperatures and high atmospheric CO_2_ levels make plants more vulnerable to bacterial infection. Moreover, Xcc enhances and accelerates the oxidative stress already triggered by climate change in oilseed rape plants. On the other hand, this study developed an effective method for the early detection of Xcc-infected oilseed rape plants using computer vision and deep learning algorithms. The classification performance of all tested algorithms (fed with parameters describing histograms obtained from regions of interest) was successful in every climatic treatment, with kNN and SVM being the best performers. However, the predictive ability of the developed models may decrease under the extreme climate conditions projected for the future. Overall, these findings highlight the importance of understanding the mechanisms underlying plant–pathogen interactions and the effects of climate change on plant physiology to develop effective strategies for managing plant diseases in a changing environment. It also emphasizes the potential of computer vision and AI classification algorithms for early disease detection of crop diseases, noting the importance of considering environmental conditions in the performance of these techniques. Thus, the results of this research could potentially be used to improve disease surveillance and precision agriculture today and in the future.

## 4. Materials and Methods

### 4.1. Plant and Bacterial Growth

Oilseed rape plants (*Brassica napus* L. var. *napobrassica*; Franchi Sementi, Grassobbio, Italy) were subjected to two types of treatments: biological and climatic treatments. Mock-controls and two races (1 and 4) of the pathogenic bacterium Xcc constituted the three biological treatments. Race 1 (HRI 3811; originally isolated from *B. oleracea* in USA) and race 4 (HRI 1279A; originally isolated from *Brassica oleracea capitata* in UK) are conserved in the Warwick University (Coventry, UK) [99]. On the other hand, three climatic treatments were selected to evaluate the impact of climate change on plants and pathogens: intermediate and extreme projections of climate change (RCP 4.5 and RCP 8.5, respectively), the CCC the control treatment (Table 2). For each experiment, plants were kept in the corresponding climatic treatment from the moment of sowing until the end of the experiment. Ambient temperature and CO_2_ concentration shown in Table 2 were selected according to the data regionalized for Castilla y León by the AEMet for CCC and those matching to RCP 4.5 and RCP 8.5 in years 2081–2100. Day and night temperatures correspond to the average values during September, the sowing, and the first stages of the growth season. In addition, the growth chamber was set to a 16/8 h day/night regime with 60% relative humidity and 200 µmol photon m^−2^ s^−1^ of photosynthetically active radiation light for each climatic treatment.

### 4.2. Inoculation of Leaves with Races 1 and 4 of Xanthomonas campestris pv. campestris and Kinetics of Bacterial Growth

When plants were approximately 3.5 weeks old, the fourth leaf was inoculated with the pathogen by clipping four secondary veins with rat tooth tweezers previously dipped in the corresponding bacterial suspension (Figure 1). To prepare this suspension, Xcc races 1 and 4 were grown for 24 h at 28 °C on LB plates (5 g/L sodium chloride; 10 g/L tryptone; 5 g/L yeast extract; 14 g/L bacteriological agar at final pH 7.0 ± 0.2) and washed with sterile 10 mM MgCl_2_; the optical density was then adjusted at 600 nm to 0.1, corresponding to 10^8^ cfu·mL^−1^. The mock control was performed by clipping the leaves with the tweezers dipped in a 10 mM MgCl_2_ solution without bacteria. At least two experiments per climatic treatment were evaluated, providing similar results.

Determination of bacterial density per leaf area was performed by extracting bacteria from six 4.15 cm^2^ leaf disks ground in 10 mM MgCl_2_. Serial dilutions of the bacteria in 10 mM MgCl_2_ were plated onto LB plates, and cfu counts were performed after 48 h, according to [100].

### 4.3. Physiological Determinations Using Biochemical Methods

For TAA, lipid peroxidation and soluble phenolic content measurements, as well as pigments determinations, six leaves were sampled per biological treatment at 6 and 10 DAI by cutting out a 4.15 cm^2^ disk concentric to an infection point. Samples were immediately frozen in liquid nitrogen and stored at −80 °C until processing. Shimadzu UV1800 spectrophotometer (Shimadzu Corporation, Tokyo, Japan) was used to perform all spectrophotometric measurements.

Colorimetric assays were performed as in [34]. Briefly, the method developed by [101] was used to determine TAA, which is based on the ability of the samples to scavenge the oxidative radical 2,2′-azinobis(3-ethylbenzothiazoline-6-sulfonate) (^−.+^) present in the spectrophotometer cuvette. The results are expressed as ascorbic acid equivalents referred to in the sampled area (nmol ascorbic acid · cm^−2^). The malondialdehyde (MDA) content obtained using the thiobarbituric acid reaction described by [102] was used to measure lipid peroxidation. Results are expressed as MDA equivalents per leaf area (nmol MDA · cm^−2^). The measurement of total soluble phenolic content was carried out using the Folin–Ciocalteu method [103,104], and results are expressed as caffeic acid equivalents referred to sampled area (µg caffeic acid · cm^−2^). Measurements of Chl, Car, and Xanth needed to calculate the ratios Chl *a*/*b* and total Chl/(Car + Xanth) were performed following the method described by [105], whereas Anth quantification was performed using the protocol described by [106].

### 4.4. Statistics

Graphs (averages ± standard errors) and statistical analysis were performed with Microsoft Office Excel 2016 (Microsoft Corporation, Redmond, WA, USA). Two-tailed Student t-test was used to compare bacterial growth, as well as biological and climatic treatments at each DAI assayed. Differences were considered significant at *p* < 0.05 (*), *p* < 0.01 (**), or *p* < 0.001 (***) and were indicated by different lowercase or uppercase letters in the corresponding figures.

### 4.5. Computer Vision

Measurements using imaging sensors were acquired on inoculated but still symptom-free leaves of oilseed rape plants at 3 (RCP 4.5 and RCP 8.5) or 6 DAI (CCC). Leaves remained attached to the plant during the whole experiment. Eleven or twelve leaves per biological treatment were sequentially imaged using three different cameras, according to [34,48].

Thermal images were acquired using a FLIR A305sc camera (FLIR Systems, Wilsonville, OR, USA). Inside de growth chamber, the thermal camera was placed vertically 50 cm above the leaves, and 10 thermal images were recorded at a rate of one image per second [27,29]. Images of leaf T were adjusted to a fixed scale using the FLIR ResearchIR v. 3.4 software and saved for further analysis.

The customized Open FluorCam FC 800-O (Photon Systems Instruments, Brno, Czechia) was used to record MCFI. Each leaf was placed 50 cm below the camera. According to [100,103], the fluorescence emission of the leaf was excited for 2 min using a 355 nm UV lamp, and F440, F520, F680, and F740 (blue, green, red, and far-red fluorescence, respectively) were sequentially acquired for 30 s using appropriate cutoff filters. FluorCam v. 7.1.0.3 software averaged nine images per fluorescence region to obtain black-and-white images of each MCFI parameter. The software then applied a fixed scale to all images and saved them. F520 images were selected for further analysis.

Hyperspectral reflectance of leaves was registered with a Pika L hyperspectral imaging camera (Resonon, Bozeman, MT, USA) in the visible (400–700 nm) to near-infrared spectral range (700–1000 nm). According to [48], dark and light corrections were performed prior to leaf measurements. Dark correction was made in darkness. For light correction, a homogenous white calibration tile provided by Resonon was illuminated with four xenon lamps, providing homogeneous light intensity between 400 and 1000 nm. The leaves were then placed on a translation stage 50 cm below the sensor. The leaves remained homogeneously illuminated while the camera composed the images. Spectronon v. 2.134 software was used for dark and light corrections, as well as to save the images of selected VIs (WBI and DBI_3_) at a fixed scale for further analysis. The equations for calculating WBI [95] and DBI_3_ [48] are as follows:

WBI = R_970_/R_900_ (1)

DBI_3_ = R_578_/R_529_ (2)

### 4.6. Image Analysis to Obtain Parameters Used in Machine Learning Classifying Algorithms

For the early diagnosis of infected plants in each climatic treatment, the images of T, F520, WBI, and DBI_3_ were chosen. The main goal was to find parameters from those images that would serve to differentiate healthy from Xcc-infected oilseed rape leaves when implemented in classifying algorithms. Four ROIs approximately 4.15 cm^2^ in extent and concentric to the clipping points practiced in the fourth leaf were selected using FIJI, a free processing package that facilitates scientific image analysis (https://imagej.net/software/fiji/ (accessed on 12 July 2022); [107]). The sample size when using ROIs for image analysis is shown in Table 3. For each ROI, the pixel value distribution as well as the parameters defining those histograms (maximum, minimum, extent = maximum − minimum, mean, mode, median, standard deviation, skewness, and kurtosis), were obtained by FIJI. For each image (T, F520, WBI, and DBI_3_), all these variables were compared by two-tailed Student t-test, and those showing significant differences between healthy and Xcc-infected leaves were organized in databases (Microsoft Excel), one per climatic treatment, and used as input data for several classifying algorithms (Table 1).

### 4.7. Plant Classification Using Machine Learning

Classification analyses were made by KNIME free version 4.5.2 (KNIME AG, Zurich, Switzerland; www.knime.com (accessed on 13 July 2022); [108]). Each database was firstly rescaled from zero to one to ensure comparison between biological treatments, according to the equation: rescaled value = (x − minimum)/maximum. Classifying algorithms were trained and then cross-validated using stratified sampling, applying the same random seed for each algorithm and 10 validations. Four models were built for each one of the three climatic treatments by analyzing the corresponding databases with four supervised classifying algorithms: ANN, SVM, LRA, and kNN. The best algorithms fit for the prediction of healthy and Xcc-infected oilseed rape leaves are provided in Table S1. Any modification of these parameters worsened the classification performance, which was evaluated against five indicators: (i) the true negative rate or specificity; (ii) the true positive rate or sensitivity; (iii) the percentage of total right guesses or accuracy; (iv) F-measure, which is the weighted harmonic mean of precision and sensitivity; where precision is the number of correct healthy samples divided by the number of all plants classified as ‘healthy’; and (v) Cohen’s kappa, which indicates the proportion of correct classifications that are not due to chance.

## Figures and Tables

**Table 1:** List of parameters defining the histograms of the selected images that have been incorporated into the databases to feed the classifiers for each climatic treatment. DBI_3_: diseased broccoli index 3; F520: green fluorescence; T: temperature; T_extent = T_maximum − T_minimum; WBI: water balance index.

CCC	RCP 4.5	RCP 8.5
F520_mean	F520_mean	F520_standard deviation
F520_standard deviation	F520_minimum	F520_skewness
F520_mode	F520_mode	F520_kurtosis
F520_median	F520_median	T_maximum
F520_skewness	F520_skewness	T_standard deviation
F520_kurtosis	F520_kurtosis	T_extent
T_maximum	T_maximum	WBI_mean
T_minimum	T_minimum	WBI_median
T_mean	T_mean	WBI_skewness
T_standard deviation	T_standard deviation	WBI_kurtosis
T_extent	T_extent	DBI_3__mean
WBI_mean	WBI_kurtosis	DBI_3__standard deviation
WBI_median	DBI_3__mean	DBI_3__median
DBI_3__skewness	DBI_3__mode	DBI_3__skewness
DBI_3__kurtosis	DBI_3__median	DBI_3__kurtosis
	DBI_3__skewness	
	DBI_3__kurtosis	

**Table 2:** Climatic treatments used in this work. CCC represents the current climatic conditions registered for September (sowing season and early stages of oilseed rape growth) in Castilla y León, the main oilseed rape growing region in Spain. RCP 4.5 and RCP 8.5 are the representative concentration pathways 4.5 and 8.5, respectively, i.e., projections of potential future climatic conditions in the same region for the years 2080–2100. Ppm: parts per million; T: temperature.

Climatic Treatment	Day T (°C)	Night T (°C)	CO_2_ Concentration (ppm)
CCC	26	12	408
RCP 4.5	29	15	650
RCP 8.5	32	18	1000

**Table 3:** Sample size (*n*) used for training and cross-validation of the classifying algorithms in each climatic treatment.

Climatic Treatment	*n* for Healthy	*n* for Xcc-Infected	Total *n*
CCC	44	88	132
RCP 4.5	48	96	144
RCP 8.5	48	92	140

## Data Availability

The original contributions presented in the study are included in the article/Supplementary Material. Further inquiries can be directed to the corresponding author.

## Supplementary Materials

The following supporting information can be downloaded at: https://www.mdpi.com/article/10.3390/plants12061322/s1. Figure S1: In vitro growth kinetics of *Xanthomonas campestris* pv. *campestris* (Xcc) measured as colony forming units per milliliter (cfu · cm^−2^) during 48 h. Bacterial density found in liquid LB medium grown under CCC (a) and RCP 4.5 (b). No bacteria could be recovered from LB at RCP 8.5. Races 1 and 4 of Xcc were cultivated in the growth chamber to ensure they were grown in the same climatic treatments as plants. The cotton plug of the tubes guaranteed proper gas exchange with the growth chamber atmosphere. In the graphs, the sequence of days and nights is indicated by white and gray rectangles, respectively. Daytime and nighttime temperatures, as well as CO_2_ concentrations, are indicated for each climatic treatment. Data represent means ± standard error of four samples. Significant differences between biological treatments are indicated by asterisks at *p* < 0.05 (*), *p* < 0.01 (**), and *p* < 0.001 (***). Table S1. Best algorithms fit for the prediction of healthy and *Xanthomonas campestris* pv. *campestris*-infected oilseed rape leaves.

## Abbreviations

ABTS^−.+^: 2:2′-azinobis(3-ethylbenzothiazoline-6-sulfonate; AEMeT: Spanish Agencia Estatal de Meteorología; Anth: anthocyanins; AI: artificial intelligence; ANN: artificial neural network; AR: Assessment Report; Car: carotenoids; CCC: current climate conditions; cfu · cm^−2^: colony forming units per square centimeter; Chl: chlorophyll; DAI: days after inoculation; DBI_3_: disease broccoli index number 3; EU: European Union; F440: blue fluorescence; F520: green fluorescence; IPCC: Intergovernmental Panel on Climate Change; kNN: k-nearest neighbors analysis; LRA: binary logistic regression analysis; MCFI: multicolor fluorescence imaging; MDA: malondialdehyde; n: sample size; ppm: parts per million; RCP: representative concentration pathway; RCP 4.5: intermediate scenario of climate change; RCP 8.5: extreme scenario of climate change; ROI: region of interest; ROS: reactive oxygen species; SVM: support vector machine: T: temperature; TAA: total antioxidant activity; VI: vegetation index; WBI: water balance index; Xanth: xanthophylls; Xcc: *Xanthomonas campestris* pv. *campestris*.
