# Supplementary material for: Assessment of Black Rot in Oilseed Rape Grown under Climate Change Conditions Using Biochemical Methods and Computer Vision
Source: Plants (Basel). 2023 Mar 14;12(6):1322. doi: 10.3390/plants12061322 (PMC10058869; doi:10.3390/plants12061322)
Supplement: Supplementary file 1 [file plants-12-01322-s001.zip › Supplementary Table.pdf]

Table S1. Best algorithms fits for the prediction of healthy and *Xanthomonas campestris* pv. *campestris*-infected oilseed rape leaves.

**Algorithm**	**Climatic treatment**		
	**CCC**	**RCP 4.5**	**RCP 8.5**
Artificial neural Networks (ANN)	Back-propagation multilayer perceptron based<br>1 hidden layer with 8 neurons<br>100 iterations maximum	Back-propagation multilayer perceptron based<br>1 hidden layer with 9 neurons<br>100 iterations maximum	Back-propagation multilayer perceptron based<br>1 hidden layer with 8 neurons<br>100 iterations maximum
Support vector machines (SVM)	Polynomial kernel<br>Power = 1<br>Bias = 1.5<br>$\gamma = 1.5$<br>Overlapping penalty = 1	Polynomial kernel<br>Power = 1<br>Bias = 1<br>$\gamma = 1$<br>Overlapping penalty = 1	Polynomial kernel<br>Power = 1<br>Bias = 1<br>$\gamma = 1$<br>Overlapping penalty = 1
Logistic regression analysis (LRA)	Stochastic average gradient<br>Epochs = 100<br>$\varepsilon = 10^{-5}$<br>Fixed learning rate<br>Step size = 0.1<br>Uniform regularization	Stochastic average gradient<br>Epochs = 100<br>$\varepsilon = 10^{-5}$<br>Fixed learning rate<br>Step size = 0.1<br>Uniform regularization	Stochastic average gradient<br>Epochs = 100<br>$\varepsilon = 10^{-5}$<br>Fixed learning rate<br>Step size = 0.1<br>Uniform regularization
k nearest neighbors (kNN)	k = 3<br>Euclidean distance	k = 4<br>Euclidean distance	k = 6<br>Euclidean distance
