# Supplementary figures and images for: Assessment of Black Rot in Oilseed Rape Grown under Climate Change Conditions Using Biochemical Methods and Computer Vision
Source: Plants (Basel). 2023 Mar 14;12(6):1322. doi: 10.3390/plants12061322 (PMC10058869; doi:10.3390/plants12061322)

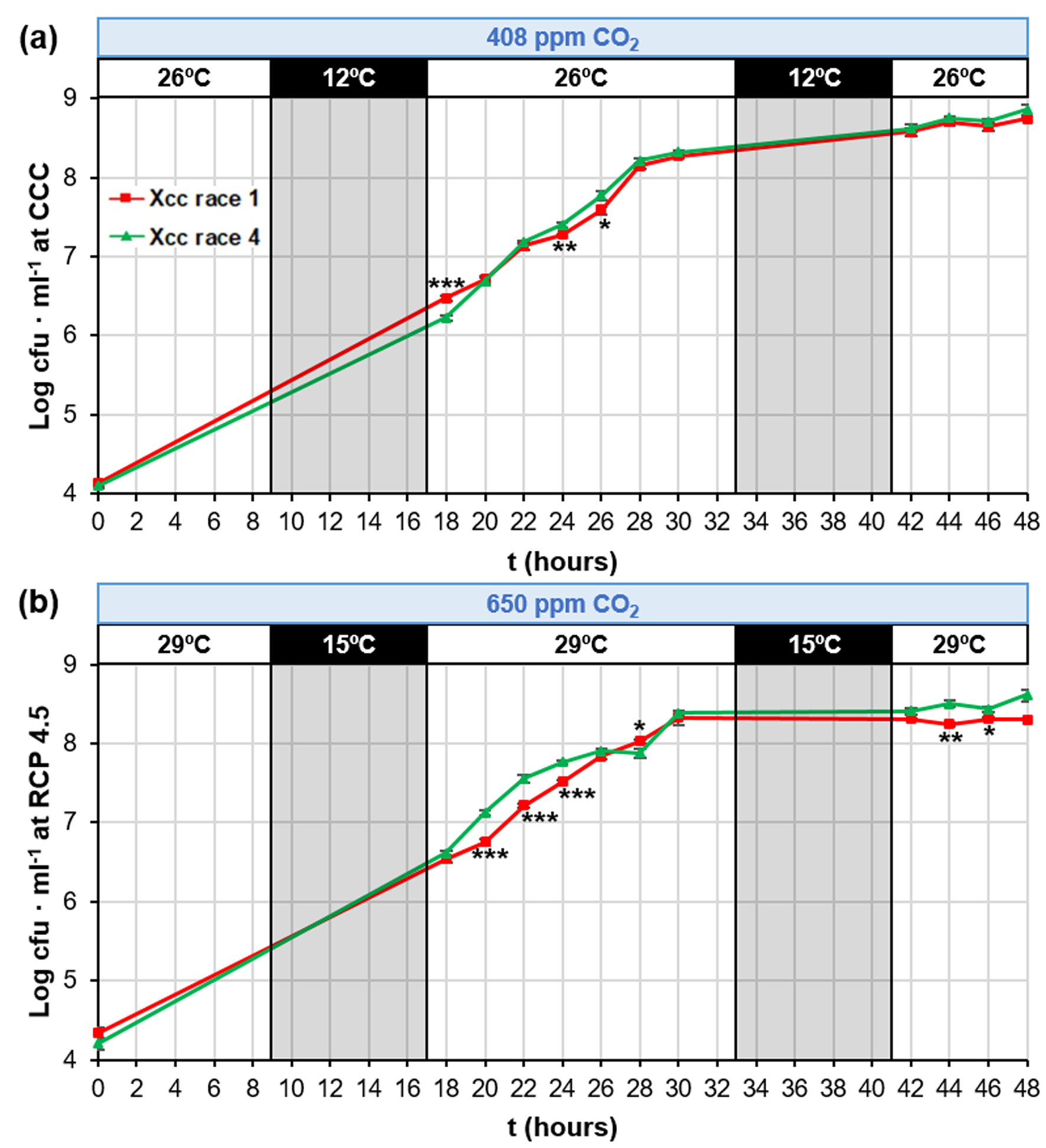

Supplement: Supplementary file 1 [file plants-12-01322-s001.zip › Supp. Fig. S1.tif]
